# HbA1c and skeletal muscle health in type 2 diabetes: phenotype-specific associations across different analytical directions and mechanistic insights

**DOI:** 10.3389/fendo.2026.1993144

**Published:** 2026-10-28

**Authors:** Zheng Lian, Yanze Liu, Guangwen Liu, Mengyuan Li, Zhen Zhong, Wenzhe Wang, Shixing Wu, Hongfeng Wang, Lin Yao

**Affiliations:** 1 College of Acupuncture and Massage, Changchun University of Chinese Medicine, Changchun, Jilin, China; 2 Acupuncture and Tuina Center, The Third Affiliated Clinical Hospital of Changchun University of Chinese Medicine, Changchun, Jilin, China; 3 Research Office, Affiliated Hospital of Changchun University of Chinese Medicine, Changchun, Jilin, China; 4 Institute of Acupuncture and Massage, Northeast Asian Institute of Traditional Chinese Medicine, Changchun University of Chinese Medicine, Changchun, Jilin, China

**Keywords:** HbA1c, HbA1c-derived glycemic exposure, muscle mass, muscle strength, physical performance, sarcopenia, type 2 diabetes mellitus

## Abstract

Sarcopenia is increasingly recognized as a clinically relevant complication of type 2 diabetes mellitus (T2DM), but its association with glycated hemoglobin (HbA1c) remains unclear. This review synthesizes evidence from 30 clinical reports examining the relationship between HbA1c-derived glycemic exposure measures and skeletal muscle outcomes in adults with T2DM, with studies categorized by muscle phenotype and analytical direction. Risk of bias was assessed using design-specific JBI checklists (lower/moderate/higher concern:11/16/3), and conclusions emphasized adjusted estimates from lower or moderate-concern studies. Available evidence indicates that the association between HbA1c-derived glycemic exposure measures and skeletal muscle health differs substantially across phenotypes. Longitudinal studies provide more consistent temporal evidence linking HbA1c trajectories to changes in muscle mass, whereas some prospective studies suggest that baseline muscle characteristics may provide prognostic information regarding subsequent glycemic outcomes. Evidence for physical performance remains inconsistent, and associations with composite sarcopenia vary according to diagnostic frameworks and phenotype composition. Potential biological mechanisms involving impaired insulin signaling, advanced glycation end product accumulation, mitochondrial dysfunction, inflammation, neuropathy, and microvascular impairment may represent biologically plausible and overlapping pathways contributing to these associations. However, direct human evidence supporting pathway-specific effects on individual muscle phenotypes remains limited. Current evidence supports a phenotype-specific interpretation of the HbA1c–skeletal muscle relationship but does not establish bidirectional causality. HbA1c should be interpreted as a marker of glycemic exposure rather than an indicator of overall skeletal muscle health, and satisfactory glycemic control does not exclude sarcopenia or impairment in individual muscle domains. Prospective studies with repeated glycemic assessments and multidimensional muscle evaluations are needed to clarify temporal relationships and clinical implications.

## 1 Introduction

Sarcopenia is a progressive skeletal muscle disorder characterized by core features of decreased muscle strength, reduced muscle mass/quality, and impaired physical performance, which represent distinct assessment domains (1). This condition is closely associated with increased risks of falls, frailty, disability, loss of independence, institutionalization, and mortality (2, 3). In patients with T2DM, factors such as aging, insulin resistance, physical inactivity, nutritional imbalance, and complications may synergistically accelerate skeletal muscle deterioration (4, 5). Multiple studies have demonstrated that declines in muscle mass and strength are more pronounced in individuals with T2DM than in those without diabetes, and that skeletal muscle impairment may in turn adversely affect peripheral glucose disposal and exacerbate insulin resistance (6–8). These findings suggest a potentially reciprocal relationship between metabolic dysfunction and skeletal muscle health; however, existing clinical studies have mainly examined these associations from separate analytical directions rather than demonstrating a bidirectional biological relationship.

HbA1c, as a commonly used indicator of chronic glycemic exposure, has been increasingly utilized in recent years to study the association between sarcopenia and its component phenotypes (9). Observational studies have reported that higher HbA1c levels or poor glycemic status are associated with decreased muscle mass, weakened muscle strength, decline in physical function, and composite sarcopenia (10, 11). Systematic reviews and meta-analyses also support the overall association between poor glycemic control in T2DM and sarcopenia (8, 10), but the reported correlations vary among different studies (11). This heterogeneity indicates that sarcopenia is not a single and homogeneous outcome. Currently, the consensus framework for sarcopenia includes the European Working Group on Sarcopenia in Older People 2 (EWGSOP2) and the Asian Working Group for Sarcopenia 2019 (AWGS 2019), which define muscle strength, muscle mass or quality, and physical function as distinct but non-interchangeable domains and have different diagnostic roles (12, 13).

In existing studies, HbA1c has been assessed using different thresholds, continuous values, or longitudinal changes, and skeletal muscle has been assessed using heterogeneous definitions and measurement techniques (14, 15). Studies also differ in analytical direction. Some evaluate HbA1c-derived glycemic exposure measures as predictors of skeletal muscle impairment (16), whereas others examine whether muscle mass or strength is associated with HbA1c-based glycemic outcomes (7, 16, 17). In addition, mechanistic studies have found that biological pathways linking chronic hyperglycemia to skeletal muscle deterioration may affect muscle quantity and function in different ways (5). These pathways include impaired insulin signaling and protein homeostasis, accumulation of advanced glycation end products (AGEs), mitochondrial dysfunction, oxidative stress, inflammation, neuropathy, and microvascular impairment (18–20). Therefore, the overall association between HbA1c and sarcopenia may obscure phenotype-specific relationships with its individual components, including muscle mass, muscle strength, and physical performance.

Accordingly, we propose a phenotype-specific framework incorporating evidence from different analytical directions to interpret the relationship between glycemic exposure and skeletal muscle health in T2DM, existing evidence summarized as shown in **Figure 1**. Rather than treating HbA1c as a direct surrogate marker of sarcopenia, this framework considers its associations separately across muscle mass, muscle strength, physical performance, and composite sarcopenia, while also recognizing that skeletal muscle impairment may adversely influence glucose metabolism. Within this framework, this structured narrative review synthesizes clinical evidence across distinct skeletal muscle phenotypes and analytical directions, distinguishing studies in which HbA1c-derived glycemic exposure measures are evaluated as exposures from those in which skeletal muscle characteristics are examined as predictors of subsequent or concurrent glycemic outcomes. We further summarize potential biological mechanisms to provide mechanistic context for the observed phenotype-specific associations, while avoiding causal interpretation beyond the available evidence.

**Figure 1:**
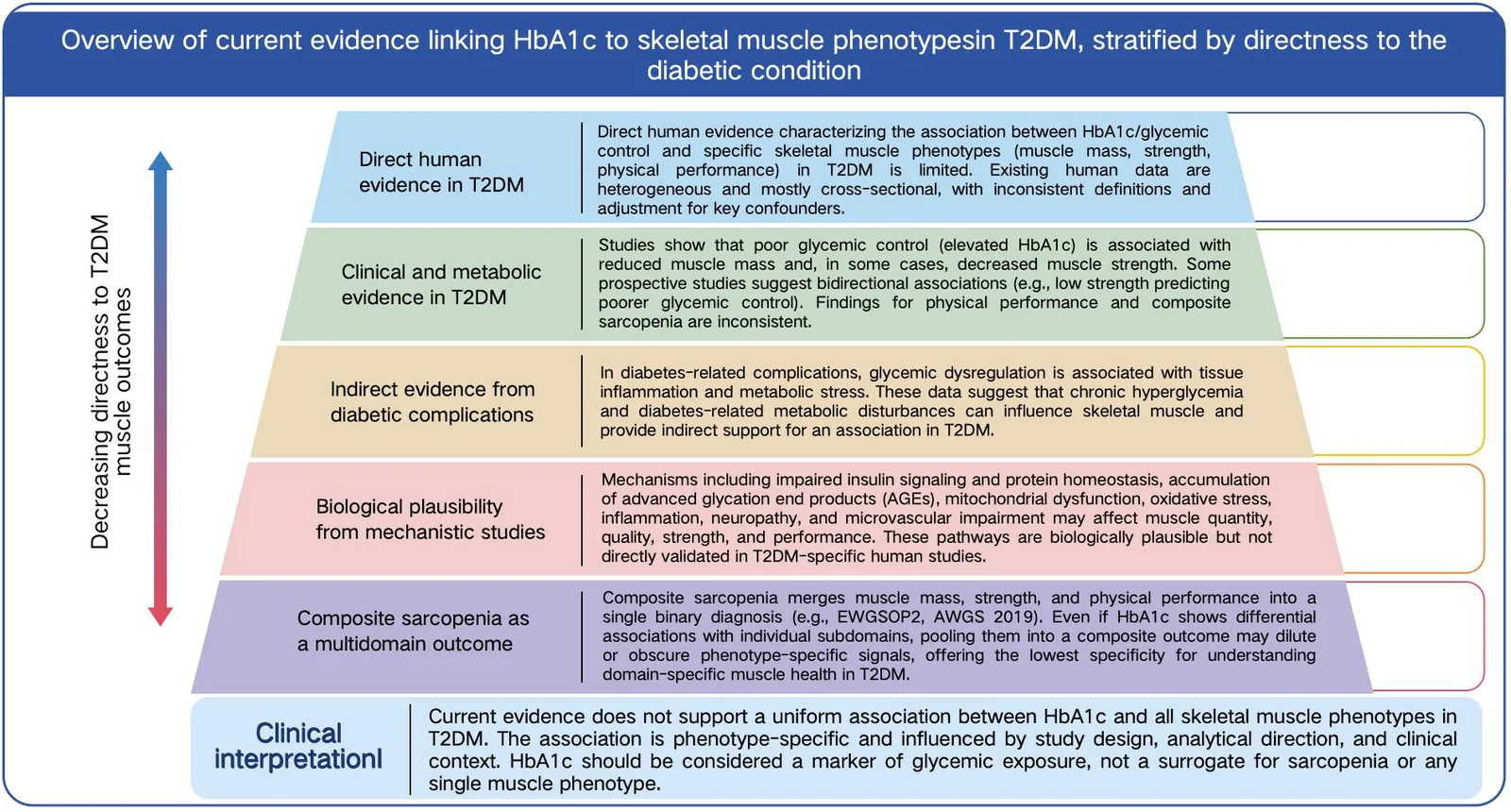
Overview of current evidence linking HbA1c to skeletal muscle phenotypes in T2DM, stratified by directness to the diabetic condition.

## 2 Materials and methods

### 2.1 Study design

This review adopts the methodological framework of structured narrative review. Due to the significant heterogeneity in the clinical and methodological aspects of the included studies, a research process was formulated, covering the search strategy, inclusion and exclusion criteria, data extraction, summary of evidence tables, and narrative synthesis based on phenotypes, in order to enhance the rigor and reproducibility of study screening and evidence interpretation. Therefore, no quantitative meta-analysis was conducted. The main evidence synthesis focused on evaluating the clinical studies assessing the association between HbA1c or glycemic exposure and skeletal muscle phenotypes in adult patients with T2DM. Mechanistic evidence was separately reviewed as a background support for biological rationality, to assist in interpreting the potential pathways underlying the clinical associations, rather than serving as direct evidence for causal inference.

### 2.2 Search strategy

PubMed and Web of Science Core Collection were searched from database inception through 31 August 2026. PubMed and Web of Science searches were restricted to English-language publications. The search strategy combined terms related to (i) HbA1c-derived glycemic exposure, (ii) sarcopenia or skeletal muscle phenotypes, and (iii) type 2 diabetes mellitus. The complete database-specific search strategies, including exact search strings, field tags, filters, execution dates, and numbers of records retrieved, are provided in **Supplementary Table S1**.

Google Scholar was used only as a supplementary source, not as a formal database. Records identified through Google Scholar were screened by title and abstract and cross-checked against the PubMed and Web of Science records. Additional potentially eligible studies were identified through backward citation searching of included original studies and relevant reviews. All retrieved records were imported into reference management software, and duplicate records were removed by automated detection followed by manual verification.

### 2.3 Eligibility criteria

Studies meeting the following criteria were eligible for inclusion: (1) The study population consisted of adults aged ≥18 years with T2DM. Studies were also eligible if the population was predominantly composed of individuals with T2DM (≥80% of the total sample) or if T2DM-specific results could be separately extracted from mixed populations; (2) HbA1c-related glycemic exposure was evaluated using one of the following approaches: (i) a single continuous HbA1c measurement, (ii) categorical HbA1c categories based on study-specific thresholds, (iii) longitudinal HbA1c change or trajectory measures, or (iv) achievement of predefined HbA1c targets during follow-up. Other glucose-related biomarkers were not considered primary exposures unless HbA1c-specific results were separately available; (3) The study assessed at least one predefined skeletal muscle outcome, including muscle mass, muscle strength, physical performance, muscle quality, or composite sarcopenia; (4) The study used observational or intervention designs and reported associations between HbA1c-related glycemic exposure and skeletal muscle outcomes, or between skeletal muscle characteristics and subsequent or concurrent glycemic outcomes.

Exclusion criteria were as follows: (1) Studies involving non-T2DM populations (e.g., type 1 diabetes, gestational diabetes, or prediabetes), or mixed diabetes populations without extractable or predominantly T2DM-specific data; (2) Studies involving children or adolescents; (3) Animal or *in vitro* studies; (4) Reviews, commentaries, case reports, or conference abstracts; and (5) Studies that only descriptively reported HbA1c and skeletal muscle-related variables without assessing their association.

### 2.4 Study selection and data extraction

Two reviewers independently screened the titles and abstracts of all unique records and subsequently assessed potentially eligible full-text reports according to the predefined eligibility criteria. Disagreements at either stage were resolved through discussion and consensus; unresolved disagreements were adjudicated by a third reviewer.

Data were independently extracted by the same two reviewers using a standardized extraction form. Extracted information included study design and population, sample size, HbA1c definition and analytical role, skeletal muscle phenotype and assessment method, sarcopenia definition when applicable, analytical direction, effect estimates with corresponding confidence intervals or *P* values, covariate adjustment including antidiabetic treatment, physical activity, body composition and nutritional variables, subgroup or sensitivity analyses, major interpretive limitations, nutritional variables, and prevalence. Discrepancies in extracted data were resolved by consensus, with unresolved differences adjudicated by a third reviewer. A third reviewer verified the final extraction table. Reports derived from the same underlying cohort were retained when they addressed distinct analytical questions, skeletal muscle phenotypes, or follow-up periods; such reports were not treated as independent replications.

### 2.5 Evidence synthesis

Clinical evidence was classified and synthesized according to distinct skeletal muscle domains, including muscle mass, muscle strength, physical performance, and composite sarcopenia. The study also classified according to the direction of analysis: (1) using HbA1c-derived glycemic exposure measures as the exposure factor or predictor for skeletal muscle outcomes, including baseline HbA1c level, HbA1c categories, longitudinal HbA1c changes, or achievement of HbA1c targets; (2) using skeletal muscle phenotypes as predictors of subsequent or concurrent glycemic outcomes, including HbA1c-based outcomes; (3) cross-sectional association analysis without a clear temporal sequence. If multiple models were reported in the research, the fully adjusted effect estimate that best matched the preset phenotype was prioritized; if no adjusted estimate was available or the unadjusted analysis was the main analysis method of the study, the unadjusted results were retained.

Data extraction covered study design, sample size, covariate adjustment, treatment-related confounding factors, analysis direction, reporting inconsistency, and cohort overlap. These methodological characteristics were all included in the narrative interpretation for consideration, rather than being transformed into formal bias risk scores. These analytical directions were interpreted as distinct evidence streams and were not considered proof of reciprocal or bidirectional biological causality.

### 2.6 Critical appraisal and risk-of-bias assessment

Clinical reports were appraised using design-specific JBI critical appraisal checklists: the analytical cross-sectional checklist (8 items) for cross-sectional or concurrent analyses and the cohort checklist (11 items) for longitudinal or cohort analyses (21, 22). Two reviewers independently applied the relevant checklist to each report. Each item was rated as yes, no, unclear, or not applicable. Disagreements were resolved by discussion and consensus; unresolved disagreements were adjudicated by a third reviewer. No aggregate numerical score was calculated because summed scores may conceal critical flaws and mask differences across domains. Instead, the four critical domains considered were exposure measurement, outcome ascertainment, confounding, and statistical analysis; for cohort studies, attrition and incomplete follow-up were also considered. Overall concern was classified as lower (all critical domains adequate), moderate (one critical domain unclear or partially inadequate, or an important design-specific concern), or higher (a critical domain clearly inadequate with additional limitations, or multiple critical concerns). Critical appraisal informed, but did not determine, study inclusion. Higher-concern studies were retained for completeness but were not used alone to support the main conclusions. Item-level ratings and quality-informed implications are provided in **Supplementary Table S2**.

## 3 Results

### 3.1 Study selection and characteristics

The database and citation searches identified 2,167 records: PubMed (n = 1,069), Web of Science Core Collection (n = 1,097), and citation searching (n = 1). After 818 duplicate records were removed (817 cross-database duplicates and 1 internal PubMed duplicate), 1,349 records were screened by title and abstract. At this stage, 1,313 records were excluded because they did not meet the eligibility criteria or were published after 31 August 2026. Thirty-six full-text reports were assessed for eligibility. Six reports were excluded: the population was not restricted to T2DM or did not provide separately extractable T2DM-specific results (n = 4), no inferential HbA1c-muscle association was reported (n = 1), and physical function was assessed only with a patient-reported quality-of-life domain rather than an objective muscle performance measure (n = 1). Thirty unique reports met the eligibility criteria and were included in the clinical evidence synthesis. The study-selection process is summarized in **Figure 2**. The included evidence was mainly from observational studies, covering cross-sectional and longitudinal studies in different geographical regions. There was considerable heterogeneity in the characteristics of participants, the definition and assessment methods of HbA1c, the measurement methods of skeletal muscle, and the diagnostic criteria for sarcopenia. As some publications were based on overlapping cohorts, the number of reports should not be interpreted as representing an equivalent number of independent study populations.

**Figure 2:**
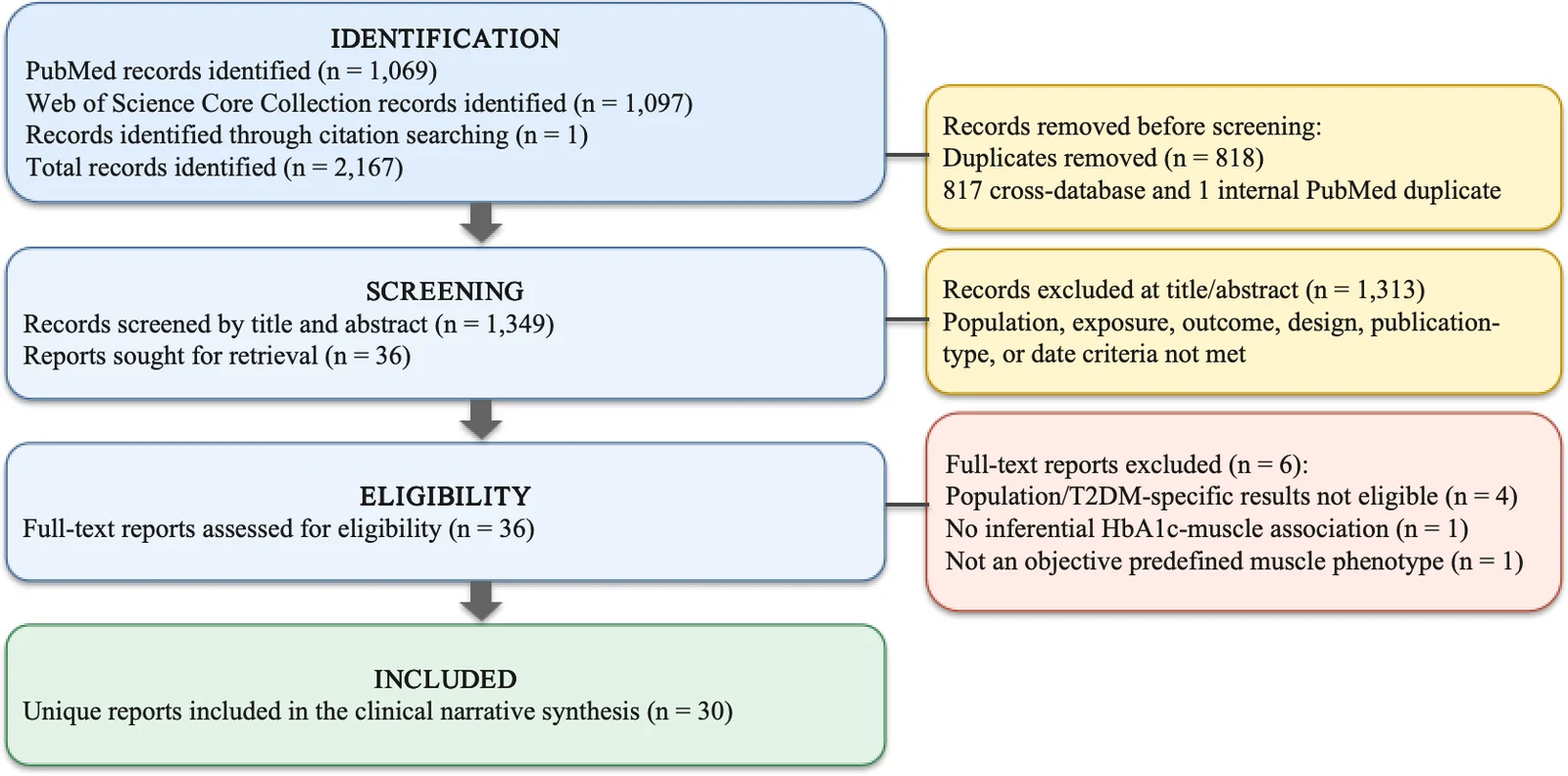
Study-selection flow diagram adapted from PRISMA 2020 for this structured narrative review.

HbA1c exposure was operationalized differently across studies, including single continuous HbA1c measurements, categorical HbA1c thresholds, longitudinal HbA1c changes, and achievement of HbA1c targets during follow-up. Notably, analytical directions differed across studies, with most studies evaluating HbA1c-derived glycemic exposure measures as exposures and skeletal muscle outcomes as dependent variables, whereas others examined whether muscle mass or strength was associated with concurrent or subsequent glycemic control. The included evidence is summarized by phenotype in **Tables 1** and **2**.

**Table 1:** Domain-specific and complementary clinical reports linking HbA1c-derived glycemic exposure measures with skeletal muscle mass, strength, and physical performance.

Report	Design/population	HbA1c definition/role	Phenotype/assessment	Analytical direction	Main effect estimate/result	Adjustment/covariates	Nutritional variables assessed/adjusted in multivariable model	Prevalence of phenotype abnormality
Shishikura et al., 2014 (Japan) (23)	Cross-sectional; n=138 T2DM	Continuous HbA1c; mean ~9.9%	Muscle mass (BIA; SMI)	Concurrent HbA1c-mass association	No significant correlation between SMI and HbA1c in either sex/age strata; multivariable model focused on stimulated C-peptide rather than HbA1c as predictor of SMI.	Correlation analyses; multivariable CPR model included SMI, sex, age, BMI, FPG, HbA1c	No direct nutritional assessment reported; BMI was included as a covariate	Not reported
Yoon et al., 2016 (Korea) (24)	Cross-sectional subsidiary cohort; 269 older men; 79 with T2DM	HbA1c categories; poor control >=8.5%	Leg muscle mass, knee-extension strength, muscle quality (strength/mass), SPPB	HbA1c to muscle quality/performance	HbA1c >=8.5% associated with decreased muscle quality: adjusted OR 4.540 (1.031-19.985), *p* = 0.045. SPPB was lower in the >=8.5% group, but no adjusted SPPB OR was reported.	Adjusted for age, BMI, smoking, alcohol, exercise	No direct nutritional assessment reported; BMI was included as a covariate	Not reported
Ozturk et al., 2017 (Turkey) (25)	Prospective observational; 147 enrolled, 105 completed; sarcopenic-obese T2DM	Baseline HbA1c >8%; follow-up groups <8% vs >=8%	Muscle mass, handgrip strength, 6-m gait speed	HbA1c category/change to domain change	In the <8% group, muscle mass increased significantly (7.42 to 7.64 kg/m2, *p* = 0.041); HGS and gait changes were NS. HbA1c-muscle mass correlations: r=-0.327, *p* = 0.039 and r=-0.183, *p* = 0.044.	Nonrandomized observational comparisons/correlations	MNA-SF and serum albumin were measured; MNA-SF did not differ between HbA1c groups at follow-up (12.3 vs 12.8, p=0.284). Neither was included in a multivariable model. No quantitative energy or protein intake was reported	Sarcopenia 100% by inclusion (EWGSOP-defined sarcopenic obesity); not a population prevalence estimate
Sugimoto et al., 2019 (Japan) (26)	Multicenter cross-sectional MUSCLES-DM; n=746 T2DM	HbA1c <6.5% ref; 6.5-<7%; 7-<8%; >=8%	BIA SMI; HGS; usual gait speed	HbA1c to domains	For HbA1c >=8% vs <6.5%: low SMI OR 5.35 (2.07-13.86), *p* < 0.001; weak HGS OR 1.95 (1.00-3.80), *p* = 0.050; slow gait OR 1.16 (0.65-2.06), *p* = 0.612.	Age, sex, BMI, exercise, albumin(nutritional marker), oral drugs, insulin, cardiovascular disease	Serum albumin was included in all multivariable models; no quantitative dietary, energy, or protein intake was assessed.	Composite sarcopenia 7.0% (52/746); modified AWGS definition using low SMI plus weak grip strength or slow gait
Nakanishi et al., 2020 (Japan) (27)	Cross-sectional; n=1,030 T2DM	Continuous HbA1c	SMI, HGS, usual gait speed	Muscle phenotype to concurrent HbA1c	SMI: beta=-0.020, *p* = 0.646; HGS: beta=-0.158, *p* = 0.0002; gait speed: beta=-0.139, *p* = 0.0001.	Multiple regression; age, sex, diabetes duration, antihypertensive and lipid-lowering medication included	No direct nutritional assessment reported	Low SMI 38.8% (400/1,030); weak grip 18.2% (187/1,030); slow gait 5.4% (56/1,030); composite sarcopenia 12.3% (127/1,030) by AWGS 2014
He et al., 2020 (China) (16)	Multicenter cross-sectional; n=1,125 T2DM	HbA1c <7% ref; 7-<10%; >=10%	Low muscle strength (EWGSOP2 probable sarcopenia); ASMI also measured	HbA1c to low strength	HbA1c 7-<10% OR 1.088 (0.675-1.755), *p* = 0.729; >=10% OR 1.396 (1.018-1.915), *p* = 0.038.	Multivariable logistic regression; age, sex, BMI, HbA1c, nephropathy, albumin and other candidate factors	Three-day dietary record measured total energy, carbohydrate, protein, and fat intake; energy/protein/fat were candidate covariates but were not retained in the final model. Serum albumin was retained and independently associated with low muscle strength. Mean intake was 29.8 kcal/kg/d and 1.13 g/kg/d protein	Low muscle strength (probable sarcopenia) 36.0% overall; 50.3% in men and 20.4% in women. Low ASMI was 11.0% versus 25.4% in men and 6.8% versus 19.1% in women without versus with probable sarcopenia (study-specific lower-quintile ASMI cutoffs)
Sugimoto et al., 2021 (Japan) (28)	Longitudinal multicenter MUSCLES-DM; n=588 T2DM	Change in HbA1c; indicator for >=1% decrease	SMI, HGS, usual gait speed	Delta HbA1c to delta domains	HbA1c decrease >=1% independently associated with SMI improvement (beta=0.113, *p* = 0.027) and gait-speed improvement (beta=0.145, *p* = 0.002), not HGS (beta=-0.005, *p* = 0.914).	Age, sex, BMI, baseline domain, baseline HbA1c, exercise, drug use, measurement site; insulin independently associated with SMI change	Nutritional status and protein intake were not assessed; the authors explicitly identify this as a limitation	Composite sarcopenia 6.3% at baseline and 7.8% after 1 year (AWGS2019; p=0.12)
Choe et al., 2021 (Korea) (29)	Cross-sectional KNHANES; n=2,498 T2DM	Good glycemic control defined as HbA1c <6.5%	Relative grip strength (HGS/BMI)	Strength to HbA1c-based glycemic outcome	Lowest vs highest grip-strength tertile: adjusted OR 0.67 (0.47-0.97) for glycemic control; middle tertile OR 0.73 (0.53-1.01).	Age, sex, education, income, smoking, alcohol, family history, obesity, hypertension, hypercholesterolemia, insulin use	No dietary variables were included in the analysis; KNHANES source data were used, but adjustment was limited to lifestyle, obesity, and clinical factors	Not reported; grip strength was analyzed by sex- and age-specific tertiles rather than a fixed low-strength threshold
Koo et al., 2021 (Korea) (17)	Prospective cohort; n=100 T2DM	Outcome: HbA1c increase >=0.6% and/or HbA1c >=9% during follow-up	HGS; BIA-derived muscle mass/body composition	Muscle phenotype to future glycemic deterioration	Low HGS HR 2.23 (1.06-4.72). In women, HR per HGS quartile 0.58 (0.37-0.93) and per muscle-mass quartile 0.46 (0.25-0.85).	Age, sex, baseline HbA1c, obesity, diabetes duration, baseline antidiabetic medication	No direct nutritional assessment reported; BMI and body composition were included as clinical covariates	Not reported; HGS was analyzed using EWGSOP thresholds and quartiles as predictors
Lin et al., 2021 (China) (30)	Longitudinal readmission cohort; n=482 T2DM	Delta HbA1c quartiles; highest quartile >=+0.61%	DXA ASM/height2	Delta HbA1c to incident low muscle mass	Among baseline non-sarcopenic patients, Q4 vs Q1 delta HbA1c OR 5.355 (1.694-16.923) for incident low-mass outcome; Q2 1.048, Q3 1.586.	Multivariable logistic regression with multiple clinical/metabolic covariates	Serum albumin was reported descriptively but was not included in the adjusted model; no dietary intake was reported	Baseline sarcopenia 35.7% (172/482); incident sarcopenia 12.6% (39/310) among baseline non-sarcopenic patients over a median of 36 months; resolution 23.8% (41/172). Study-defined ASM/height2 criterion without strength or performance components
Hiromine et al., 2022 (Japan) (31)	Cross-sectional multicenter MUSCLES-DM; n=755 T2DM (reported within a mixed diabetes cohort)	HbA1c >=8% vs <8%	BIA ASM, HGS, gait speed	HbA1c to domains	Age- and sex-adjusted model: low ASM OR 1.26 (95% CI 0.80–1.97), *P* = 0.02; low HGS OR 2.05 (95% CI 1.42–2.95), *P* < 0.001; slow gait speed OR 1.53 (95% CI 1.07–2.19), *P* = 0.02. After additional adjustment for insulin treatment, low HGS remained significant (OR 1.74, 95% CI 1.17–2.61, *P* = 0.006), whereas slow gait speed was no longer significant (OR 1.39, 95% CI 0.94–2.06, *P* = 0.10)	Age/sex; models additionally adding diabetes type, C-peptide or insulin	Serum albumin and BMI <18.5 kg/m2 were assessed. Albumin was included in separate sarcopenia and handgrip-strength models, but not in the extracted HbA1c-specific models	Among patients with T2DM aged >=65 years: composite sarcopenia 8.1% and low HGS 15.5% by AWGS2019. Low ASM and slow gait were reported graphically by age group
Alfaro-Alvarado et al., 2023 (Mexico) (32)	Cross-sectional; n=356 older adults with T2DM	Poor control HbA1c >=7.5%	Low muscle mass, low strength, low gait speed; EWGSOP2	HbA1c to domains	Age/sex-adjusted: low mass OR 1.73 (1.06-2.82), *p* = 0.027; low strength OR 1.45 (0.92-2.28), *p* = 0.107; low gait OR 1.18 (0.76-1.83), *p* = 0.462.	Age/sex plus sensitivity models including malnutrition status, frailty, comorbidities, complications, physical activity, insulin use, heart disease, nephropathy, and diabetes duration	MNA-defined malnutrition was entered in sensitivity models; the HbA1c-sarcopenia association attenuated after adjustment (p=0.098). Serum albumin was also measured. No quantitative energy or protein intake was reported	Composite sarcopenia 45.5% (162/356) by EWGSOP2; component-specific prevalence was not reported
Alabadi et al., 2023 (Spain) (33)	Cross-sectional; n=100 adults >65 y with T2DM	Continuous HbA1c; mean 7.4%	BIA FFMI, skeletal muscle mass and SMI; strength/performance	Concurrent mass-glycemic association	Women with low FFMI/SMM/SMI had HbA1c ~7.8-7.9% vs 7.2%. Adjusted HbA1c: low FFMI Exp(B)=0.908, *p* = 0.821; low SMM Exp(B)=0.495, *p* = 0.131.	Female multivariable logistic models with clinical/body-composition variables	MNA, FFQ plus 24-hour recall for protein intake, and serum albumin were assessed. Lower protein intake was observed in the low-FFMI group (18.2% vs 20.7% of energy), but nutritional variables were not retained in the final model; total energy intake was not reported	Low lean or muscle mass was defined as the sex-specific lowest quartile (approximately 25% by design), not a consensus diagnostic threshold
Nomura et al., 2024 (Japan) (6)	Longitudinal observational; n=380 T2DM	Continuous delta HbA1c	BIA skeletal muscle mass/SMI	Change-to-change association	Delta HbA1c negatively associated with change in muscle mass: standardized beta=-0.18, *p* = 0.047; HbA1c fell from 8.0+/-1.9% to 6.9+/-0.8%.	Multiple linear regression including body-composition change variables	No detailed dietary intake was measured; diet therapy was provided clinically but was not included in the model	Not reported; SMI was analyzed as a continuous change outcome
Nakanishi et al., 2024 (Japan) (7)	Prospective observational; n=233 T2DM	Outcome: achievement of HbA1c <7%	Baseline HGS tertiles	Strength to future glycemic control	Middle vs low HGS tertile HR 2.117 (1.142-3.924), *p* = 0.017; high vs low HR 4.670 (2.526-8.632), *p* < 0.0001.	Cox models with HGS and waist circumference; additional clinical covariates evaluated	No dietary intake was measured. Dietary and exercise guidance was mentioned clinically but was not included in the model	Not reported; handgrip strength was analyzed by sex-specific tertiles
Wang et al., 2026 (China) (34)	Cross-sectional; n=1,009 T2DM	Continuous HbA1c	DXA-derived SMI	Concurrent HbA1c-mass association	Men: beta=-0.062 (95% CI -0.123 to -0.001), *p* = 0.045; women: beta=-0.023 (-0.068 to 0.022), *p* = 0.325.	Sex-specific multivariable linear regression with metabolic/inflammatory variables	Serum albumin and CRP/albumin ratio were included in multivariable models; CRP/albumin was associated with low muscle mass in men. No quantitative energy, protein, or dietary-pattern assessment was reported	Low muscle mass 20.04% in men (110/549), 13.48% in women (62/460), and 17.05% overall (172/1,009; derived from reported counts) by AWGS DXA cutoffs (<7.0/<5.4 kg/m2)
Li et al., 2026 (China) (8)	Retrospective cohort analysis; n=651 T2DM	Poor control HbA1c >=7%; HbA1c also modeled continuously	CT skeletal muscle area/density at T8; IMAT and fat depots	Muscle/body composition to glycemic control	Highest vs lowest skeletal-muscle-area quartile: fully adjusted OR 0.61 (0.32-1.17), *p* = 0.142; *p*-trend=0.129. Crude trend was significant; selected subgroups showed stronger inverse associations.	Model 3: age, sex, diabetes duration, hypertension, smoking, alcohol, FBG and lipids	No direct nutritional assessment reported; body-composition measures were used instead	Not reported; CT muscle area was analyzed by quartiles without a consensus low-mass threshold
Lu et al., 2026 (China) (35)	Retrospective cross-sectional; n=838 T2DM	Continuous HbA1c	SMI, HGS, gait speed	Concurrent HbA1c-domain association	Final multivariable domain models did not retain HbA1c as an independent determinant of SMI, HGS or gait speed. In composite logistic model, HbA1c OR 0.969 (0.847-1.109), *p* = 0.646.	Sex-stratified multivariable models including age, BMI, albumin(nutritional marker), complications, therapies and other clinical variables	Serum albumin was included in multivariable models, and low albumin was associated with sarcopenia in men. Dietary intake, protein intake, and nutritional screening scores were not measured; the authors identify this as a limitation.	Sarcopenia 19.69% (165/838); low muscle mass 25.18%; low HGS 38.78%; low gait speed 10.38% by AWGS2019
Kuistono et al., 2026 (Indonesia) (36)	Descriptive-analytic cross-sectional; n=30 T2DM	HbA1c 6.6-14.0%; dichotomized at sample mean 9.54%	30-s chair-stand (leg strength/performance); sit-and-reach flexibility	HbA1c group to physical-function outcomes	Lower-HbA1c group had 2.53 more chair-stand repetitions (95% CI 1.03-4.03), *p* = 0.0018; flexibility difference 2.70 cm (0.04-5.36), *p* = 0.0466.	Unadjusted independent t tests	No direct nutritional assessment or dietary intake data were analyzed	Not reported; chair-stand performance and flexibility were analyzed as continuous outcomes
Fuso et al., 2012 (Italy) (37)	Cross-sectional comparative study; 75 non-smoking adults with T2DM without pulmonary or cardiac disease; 40 healthy controls	Continuous glycated hemoglobin; HbA1c was entered in the multiple regression model	Respiratory muscle strength and endurance; maximal inspiratory and expiratory pressures (MIP/MEP) and maximum voluntary ventilation (MVV)	HbA1c to respiratory muscle strength/endurance	MIP and MVV were lower in T2DM than in controls. In multiple regression, HbA1c was independently associated with lower MIP (beta=-0.245, P = 0.047); no independent HbA1c association with MVV was reported.	Multiple regression including age and total lung capacity for MIP; male sex and diabetic complications for MVV.	No direct nutritional assessment or quantitative dietary intake was reported.	Not reported
Yang et al., 2024 (China) (38)	Retrospective single-arm real-world cohort; 101 older adults with T2DM	Continuous baseline HbA1c; mean 7.28% in men and 7.15% in women	Skeletal muscle mass; CT at T12 with change in skeletal muscle index (SMI)	Baseline HbA1c to change in SMI after remote resistance exercise	Baseline HbA1c was positively associated with change in SMI in men (coefficient 0.208, 95% CI 0.164-0.251, P<0.001) and women (coefficient 0.349, 95% CI 0.320-0.378, P<0.001). Higher baseline HbA1c predicted greater SMI gain, but the single-arm pre-post design limits causal interpretation.	Sex-specific stepwise multiple linear regression. The male model retained baseline HbA1c and diabetes duration; the female model retained baseline HbA1c and HDL cholesterol.	No direct nutritional assessment or quantitative energy/protein intake was reported; BMI and body-composition variables were available.	Not reported

**Table 1** includes 21 reports derived from independent or overlapping datasets. Analytical direction is shown explicitly because reports modeling HbA1c as an exposure are not equivalent to those modeling skeletal muscle phenotypes as predictors of glycemic control. Reports originating from overlapping cohorts should not be interpreted as fully independent replications. For reports involving mixed diabetes populations, only data specific to T2DM were extracted and considered when available. For reports presenting multiple regression models, effect estimates were extracted from the model specified in the table, and the corresponding ORs, confidence intervals, and P values were verified against the original publication. Nutritional variables were extracted when reported; most studies assessed nutritional status using clinical indicators rather than quantitative dietary intake, and direct measures of energy or protein intake were rarely available. Fuso et al. assessed respiratory muscle strength and is therefore reported as a complementary muscle-strength phenotype rather than as a directly comparable limb-sarcopenia measure. Yang et al. was a single-arm pre-post study; its positive baseline HbA1c-to-SMI-change association is exploratory.

AGEs, advanced glycation end products; ASM, appendicular skeletal muscle; ASMI, appendicular skeletal muscle index; BIA, bioelectrical impedance analysis; BMI, body mass index; CI, confidence interval; CPR, C-peptide reactivity; CT, computed tomography; DXA, dual-energy X-ray absorptiometry; EWGSOP2, European Working Group on Sarcopenia in Older People 2; FBG, fasting blood glucose; FFMI, fat-free mass index; FPG, fasting plasma glucose; HGS, handgrip strength; IMAT, intermuscular adipose tissue; KNHANES, Korea National Health and Nutrition Examination Survey; MUSCLES-DM, Muscle Mass in Diabetes Study; NS, not significant; OR, odds ratio; SMI, skeletal muscle mass index; SPPB, Short Physical Performance Battery; CRP/Alb, C-reactive protein-to-albumin ratio; FFQ, food-frequency questionnaire; MNA, Mini Nutritional Assessment; MNA-SF, short-form Mini Nutritional Assessment.

**Table 2:** Clinical reports on composite sarcopenia and sarcopenic phenotypes in relation to HbA1c-derived glycemic exposure.

Report	Design/population	HbA1c definition/role	Composite definition/components	Analytical direction	Main effect estimate/result	Adjustment/covariates	Nutritional variables assessed/adjusted in multivariable model	Prevalence of composite sarcopenia
Trierweiler et al., 2018 (Brazil) (39)	Cross-sectional; 83 T2DM + 83 controls	Continuous glycated hemoglobin; T2DM mean 8.08+/-1.85%	FNIH-based pre-sarcopenia/sarcopenia; DXA mass + HGS	Concurrent HbA1c-composite association	Within T2DM, glycemic control (glycated hemoglobin) and chronic complications were not associated with pre-sarcopenia/sarcopenia (*p*>0.05).	Group comparisons and multivariable models for other sarcopenia determinants	Healthy/unhealthy diet was classified by fruit and vegetable servings/day, and calcium intake was assessed. The healthy-diet variable was included in the multivariable sarcopenia model. No quantitative energy or protein intake was reported.	Sarcopenia 16.2% (13 patients) and pre-sarcopenia 21% by FNIH; low muscle strength 30.1% (25/83).
Fung et al., 2019 (Singapore) (40)	Cross-sectional primary-care cohort; n=387 T2DM	Most recent single HbA1c within 6 months	AWGS sarcopenia	HbA1c to composite sarcopenia	HbA1c OR 0.81 (0.63-1.04), *p* = 0.093.	Multivariable logistic regression including demographic, clinical, socioeconomic and lifestyle variables	Frequency of protein food groups (legumes/lentils, meat/seafood/eggs, nuts/soy, dairy, and other sources) and supplement use were assessed. Dietary factors were significant only in univariable analyses and were not retained after multivariable adjustment. No energy or protein quantity was reported.	Sarcopenia 27.4%, severe sarcopenia 3.9%, and pre-sarcopenia 30.5%; low muscle mass 57.9%, low strength 31.3%, and low gait speed 9.6% using AWGS criteria.
Sugimoto et al., 2019 (Japan) (26)	Multicenter cross-sectional MUSCLES-DM; n=746 T2DM	HbA1c <6.5% ref; 6.5-<7%; 7-<8%; >=8%	Sarcopenia = low SMI plus weak HGS or slow gait	HbA1c to composite sarcopenia	Fully adjusted ORs vs <6.5%: 4.30 (1.11-16.65), *p* = 0.035; 4.48 (1.24-16.17), *p* = 0.022; 7.65 (1.95-30.00), *p* = 0.003 across increasing HbA1c categories.	Basic model: age, sex, BMI, exercise, albumin, oral drugs, insulin, cardiovascular disease; fully adjusted adds diabetes duration and retinopathy	Serum albumin was included in all multivariable models; no quantitative dietary, energy, or protein intake was assessed.	Composite sarcopenia 7.0% (52/746); modified AWGS definition using low SMI plus weak grip strength or slow gait.
Cui et al., 2020 (China) (41)	Cross-sectional; n=132 older T2DM	Continuous HbA1c	AWGS sarcopenia: low ASMI plus low HGS and/or gait speed	Concurrent HbA1c-composite association	HbA1c 7.5 (6.9-10.1)% in sarcopenia vs 8.1 (7.1-9.6)% in non-sarcopenia, *p* = 0.771; HbA1c did not enter the final independent-factor model.	Final logistic model retained age, trunk fat mass, FT4, BMI, exercise, sex, metformin, trunk skeletal muscle mass	Self-reported high-protein diet (yes/no) was assessed; it did not differ by sarcopenia status and was not retained in the final model. No quantity was reported.	Sarcopenia 28.8% (38/132); 35.6% in men and 23.3% in women by AWGS 2014.
Nakanishi et al., 2020 (Japan) (27)	Cross-sectional; n=1,030 T2DM	Continuous HbA1c	Sarcopenia severity categories: no sarcopenia, 2-factor, 3-factor groups based on SMI/HGS/gait	Composite severity to concurrent HbA1c	HbA1c differed across sarcopenia-severity categories after adjustment (trend *p* = 0.013); domain regression showed HGS and gait, not SMI, associated with HbA1c.	ANCOVA/multiple regression with age, sex, diabetes duration and vascular-risk medication covariates	No direct nutritional assessment reported.	Low SMI 38.8% (400/1,030); weak grip 18.2% (187/1,030); slow gait 5.4% (56/1,030); composite sarcopenia 12.3% (127/1,030) by AWGS 2014.
Pechmann et al., 2020 (Brazil) (42)	Cross-sectional; 177 T2DM + 146 controls	Continuous HbA1c	FNIH sarcopenia; DXA ALM/BMI + HGS; gait measured in T2DM	Concurrent HbA1c-composite association	Within T2DM, HbA1c 7.9+/-1.5% with sarcopenia vs 8.5+/-6.1% without sarcopenia, *p* = 0.41.	Unadjusted HbA1c group comparison; multivariable sarcopenia models focused on albuminuria, body fat and other factors	Unhealthy diet (<6 fruit and vegetable servings/day), calcium intake, and supplement use were assessed. Unhealthy diet was more frequent in sarcopenia (65.2% vs 41.9%) but was not retained after multivariable adjustment. Daily protein intake was not measured.	FNIH sarcopenia 12.9% (23/177); low muscle mass 27.3%; low HGS 25.9% (46/177); low gait speed 15.0% among those assessed.
de Freitas et al., 2020 (Brazil) (43)	Cross-sectional; n=242 T2DM	Continuous HbA1c	EWGSOP1 and EWGSOP2; BIA mass, HGS, TUG performance	Concurrent HbA1c-composite association	EWGSOP1: HbA1c ~8.0+/-1.6% with vs 7.8+/-1.5% without sarcopenia, *p* = 0.35; EWGSOP2: 7.9+/-1.9% vs 7.8+/-1.5%, *p* = 0.63.	Group comparisons; Poisson multivariable models for sarcopenia determinants	A validated FFQ measured energy, macronutrients, energy-adjusted nutrients, protein sources, and serum albumin. Nutritional results were descriptive and were not included in the final sarcopenia models.	Sarcopenia 21% overall; 16.9% by EWGSOP1 and 7.0% by EWGSOP2. Component abnormalities were 21.9% low HGS, 8.3% low muscle mass, and 7.4% low physical performance.
Hiromine et al., 2022 (Japan) (31)	Cross-sectional multicenter; n=755 T2DM (reported within a mixed diabetes cohort)	HbA1c >=8% vs <8%	AWGS2019: low ASM plus low HGS or slow gait	HbA1c to composite sarcopenia	Age/sex-adjusted composite OR 1.75 (0.99-3.07), *p* = 0.05; after insulin adjustment OR 1.43 (0.77-2.67), *p* = 0.26.	Age/sex with additional models adding diabetes type, C-peptide or insulin	Serum albumin and BMI <18.5 kg/m2 were assessed. Albumin was included in separate sarcopenia and handgrip-strength models, but not in the extracted HbA1c-specific models.	Among patients with T2DM aged >=65 years: composite sarcopenia 8.1% and low HGS 15.5% by AWGS2019. Low ASM and slow gait were reported graphically by age group.
Alfaro-Alvarado et al., 2023 (Mexico) (32)	Cross-sectional; n=356 older adults with T2DM	Poor control HbA1c >=7.5%	EWGSOP2 composite sarcopenia	HbA1c to composite sarcopenia	Age/sex-adjusted OR 1.79 (1.14-2.81), *p* = 0.011. Association attenuated in models adding malnutrition (*p* = 0.098), frailty (*p* = 0.073) or insulin use (*p* = 0.179), but persisted in several other sensitivity models.	Age/sex plus separate sensitivity models for comorbidity, nutrition/frailty, complications, activity, insulin, cardiac/renal disease, duration	MNA-defined malnutrition was entered in sensitivity models; the HbA1c-sarcopenia association attenuated after adjustment (p=0.098). Serum albumin was also measured. No quantitative energy or protein intake was reported.	Composite sarcopenia 45.5% (162/356) by EWGSOP2; component-specific prevalence was not reported.
Shahi et al., 2023 (India) (44)	Cross-sectional; n=229 T2DM	Continuous HbA1c; mean ~7.57%	AWGS-based sarcopenia; DXA ASMI, HGS, SPPB/chair stand	Concurrent HbA1c-composite association	No association was found between HbA1c and sarcopenia; HbA1c was included among candidate multivariable determinants but was not retained as an independent factor.	Multivariable logistic models included age, sex, diabetes duration, BMI, waist, body fat, OHA burden, complications, HbA1c, CRP and other candidates	Lower serum albumin was included in multivariable models and was independently associated with low handgrip strength and sarcopenia. Protein and calorie intake were not measured.	Sarcopenia 18.8% (43/229), possible sarcopenia 43%, and severe sarcopenia 6.1%; low HGS 16.2%, low ASMI 33%, and poor physical performance 39.3% using AWGS with SPPB/chair-stand assessment.
Sravya et al., 2023 (India) (45)	Cross-sectional case-control; 159 T2DM + 79 controls	HbA1c <7% vs >=7%	AWGS2019; DXA muscle mass, HGS, 6-m gait speed	HbA1c to composite sarcopenia within T2DM	Univariate OR for HbA1c >=7% vs <7%: 0.788 (0.317-1.956), *p* = 0.607; HbA1c was not retained as a significant predictor after multivariable analysis.	Multivariable logistic regression; final significant predictors included BMI, physical activity, hypertension, neuropathy, serum calcium	Serum albumin was measured but was not significantly associated with sarcopenia and was not retained. High-protein nutritional supplements were excluded, and protein intake was not quantified.	AWGS2019 sarcopenia 22.0% (35/159) and severe sarcopenia 11.9%; AWGS2014 sarcopenia 17.6%. Low AMI 23.2%; low HGS 19.4% versus 16.3% and low gait speed 40.8% versus 13.2% using AWGS2019 versus AWGS2014 criteria.
Shen et al., 2026 (China) (46)	Cross-sectional single-center; n=81 T2DM	Continuous HbA1c; model cut-point >7.05% for risk score	AWGS sarcopenia; ASM, HGS, gait speed	HbA1c to composite sarcopenia	Multivariable HbA1c OR 1.459 (1.088-2.062), *p* = 0.0188; HbA1c >7.05% used in the preliminary nomogram.	LASSO selection followed by multivariable logistic regression; final predictors included HDL-C, HbA1c, height, BMI	Serum albumin differed between groups and had univariate predictive value, but it was not retained by LASSO or in the final model. No dietary intake was measured.	Sarcopenia 35.8% (29/81) by AWGS2019.
Barak et al., 2026 (Israel) (47)	Cross-sectional; n=100 community-dwelling older adults with T2DM	HbA1c as metabolic marker/risk-index component	Sarcopenic obesity severity clusters based on appendicular lean mass, HGS, knee extension, chair stand and obesity criteria	HbA1c across SO severity clusters	HbA1c: severe 7.55+/-1.16%, moderate 7.74+/-1.33%, mild 7.08+/-0.76%; F = 2.97, *p* = 0.05. HbA1c was also one component of a 5-variable SO risk index (AUC = 0.87).	Cluster analyses; ANCOVA for some secondary outcomes adjusted for age, BMI, HbA1c	A 24-hour recall assessed energy intake, protein intake in g/kg/day, and Mediterranean diet score. No significant differences were found across sarcopenic-obesity clusters, and dietary variables were not included in the multivariable models.	Sarcopenic obesity 72%; altered muscle function 1% by HGS, 49% by knee extensor strength, and 92% by 30-second chair-stand. ESPEN/EASO criteria; not directly comparable with standard sarcopenia prevalence.
Lu et al., 2026 (China) (35)	Retrospective cross-sectional; n=838 T2DM	Continuous HbA1c	Composite sarcopenia plus SMI, HGS and gait-speed domains	Concurrent HbA1c-composite association	Composite logistic model: HbA1c OR 0.969 (0.847-1.109), *p* = 0.646; sex-specific composite models also NS.	Multivariable logistic model including age, diabetes duration, albumin, ALP, creatinine, FBG, BMI, complications and therapies	Serum albumin was included in multivariable models, and low albumin was associated with sarcopenia in men. Dietary intake, protein intake, and nutritional screening scores were not measured; the authors identify this as a limitation.	Sarcopenia 19.69% (165/838); low muscle mass 25.18%; low HGS 38.78%; low gait speed 10.38% by AWGS2019.

**Table 2** includes 14 reports. Five reports also appear in **Table 1** (Sugimoto 2019; Nakanishi 2020; Hiromine 2022; Alfaro-Alvarado 2023; Lu 2026). Across both tables, a total of 30 unique publications/reports were identified; however, these reports include overlapping cohorts and should not be interpreted as 30 independent study populations. In particular, three reports were derived from the MUSCLES-DM cohort (26, 28, 31), and the two Nakanishi reports (7, 27) may use overlapping outpatient clinical datasets. Composite sarcopenia definitions vary across FNIH, AWGS/AWGS2019, EWGSOP2, and study-specific constructs and should not be treated as interchangeable.

ALM, appendicular lean mass; ALP, alkaline phosphatase; ANCOVA, analysis of covariance; ASM, appendicular skeletal muscle; ASMI, appendicular skeletal muscle index; AUC, area under the curve; AWGS, Asian Working Group for Sarcopenia; BIA, bioelectrical impedance analysis; BMI, body mass index; CI, confidence interval; CRP, C-reactive protein; DXA, dual-energy X-ray absorptiometry; EWGSOP1, European Working Group on Sarcopenia in Older People 1; EWGSOP2, European Working Group on Sarcopenia in Older People 2; FBG, fasting blood glucose; FNIH, Foundation for the National Institutes of Health; HGS, handgrip strength; HDL-C, high-density lipoprotein cholesterol; LASSO, least absolute shrinkage and selection operator; MUSCLES-DM, Muscle Mass in Diabetes Study; NS, not significant; OHA, oral hypoglycemic agent; OR, odds ratio; SMI, skeletal muscle mass index; SO, sarcopenic obesity; SPPB, Short Physical Performance Battery; TUG, Timed Up and Go test; CRP/Alb, C-reactive protein-to-albumin ratio; FFQ, food-frequency questionnaire; MNA, Mini Nutritional Assessment; MNA-SF, short-form Mini Nutritional Assessment.

### 3.2 Risk-of-bias assessment

Risk of bias was assessed for all 30 clinical reports. Twenty-three reports were appraised with the analytical cross-sectional/comparative checklist and seven with the cohort checklist. Overall, 11 studies were classified as lower concern, 16 as moderate concern, and 3 as higher concern (**Supplementary Table S2**). The most frequent concerns were incomplete adjustment for antidiabetic treatment, physical activity, nutritional status, body composition, and diabetes duration; selection effects related to retrospective or clinic-based sampling; and incomplete reporting or handling of attrition. Nutritional factors were assessed in some studies using variables such as serum albumin, malnutrition screening measures, or clinical nutritional indicators (16, 25, 26, 30–35, 39–47); however, detailed dietary intake data, including quantitative energy or protein intake, were rarely reported. When available, nutritional variables were incorporated as covariates in multivariable models, although the extent and methodology of nutritional assessment varied substantially across studies. These factors may represent not only baseline confounders but also time-varying determinants in longitudinal studies, because changes in HbA1c may occur concurrently with changes in glucose-lowering therapy, body weight, nutritional status, physical activity, and body composition. Several cross-sectional HbA1c-specific estimates were unadjusted or were not retained in the final multivariable model, and these results were given less weight than adjusted estimates. Among longitudinal studies, prospective designs with clear baseline exposure ascertainment provided stronger temporal support, whereas substantial attrition or change-to-change analyses limited causal interpretation. All phenotype-specific interpretations below were therefore conditioned on these quality domains; estimates from higher-concern studies were not used alone to support evidence consistency.

### 3.3 HbA1c and skeletal muscle mass

Prevalence of low muscle mass varied according to the diagnostic framework and measurement method. In studies using AWGS-based definitions, low muscle mass was reported in 17.0-57.9% of participants (27, 34, 35, 40, 44, 45). EWGSOP2-defined low muscle mass was 8.3% in one cohort of older adults with T2DM (43), whereas FNIH-defined pre-sarcopenia was reported in 21% of another cohort (39). One study defined low lean or muscle mass according to the sex-specific lowest quartile, producing an approximately 25% prevalence by design rather than by a consensus diagnostic threshold (33). These estimates therefore provide clinical context but should not be directly compared across definitions.

Evidence relating HbA1c to muscle mass depended strongly on the exposure construct and study design. In lower or moderate-concern longitudinal studies, changes in HbA1c exposure were associated with concurrent changes in muscle mass, particularly when analyses examined within-person change rather than a single HbA1c measurement (6, 28). However, these longitudinal associations should be interpreted cautiously because changes in HbA1c may occur simultaneously with changes in antidiabetic treatment, body weight, nutritional status, physical activity, and other body-composition-related factors. Although several studies adjusted for nutritional indicators, most available measures reflected nutritional status rather than direct dietary intake, limiting evaluation of the potential contribution of energy or protein intake to muscle changes. By contrast, adjusted cross-sectional associations were heterogeneous, as some studies reported inverse associations (26, 34) while others found weak or non-significant associations after adjustment for body composition and clinical factors (23, 27, 33, 35). Differences in BIA, DXA, and CT-derived measures further limited direct comparison (8, 26, 30, 34).

One additional single-arm retrospective study found that higher baseline HbA1c was associated with greater improvement in CT-derived skeletal muscle index after a remote resistance-training program (38). This positive direction contrasts with inverse cross-sectional associations and may reflect regression to the mean, selection for training, and greater initial muscle impairment among participants with higher baseline HbA1c; the absence of an untreated comparator precludes causal interpretation.

Taken together, the more consistent signal was observed for longitudinal changes in HbA1c in relation to changes in muscle mass among lower or moderate-concern studies. This evidence does not support the broader conclusion that muscle mass is intrinsically the most HbA1c-sensitive phenotype. The apparent difference between muscle mass and other phenotypes may partly reflect the fact that muscle mass was more often analyzed as a longitudinal change outcome.

### 3.4 HbA1c and muscle strength

Low muscle strength was also common in the included cohorts. AWGS-based reports identified low handgrip strength in 15.5-38.8% of participants (27, 31, 35, 40, 44, 45). One report using EWGSOP2 thresholds found low handgrip strength in 21.9% (43), and an FNIH-based report found low handgrip strength in 25.9% (42). In a large study using study-specific lower-quintile cutoffs, probable sarcopenia based on low muscle strength was present in 36.0% overall, including 50.3% of men and 20.4% of women (16). Threshold choice had a substantial effect: in one cohort, the prevalence of low handgrip strength increased from 16.3% to 19.4% when AWGS 2014 was replaced by AWGS 2019 criteria (45). These findings indicate a substantial burden of impaired strength without suggesting that estimates across definitions are interchangeable. Among lower or moderate-concern prospective studies, lower baseline handgrip strength predicted subsequent poorer glycemic control or a lower likelihood of achieving glycemic targets (7, 17). These studies provide direction-specific evidence that muscle strength may precede later changes in glycemic status, but they do not demonstrate biological bidirectionality.

In the opposite analytical direction, associations between HbA1c and prevalent strength were heterogeneous. Some studies identified weaker strength or poorer muscle quality at higher HbA1c levels (16, 24, 26, 31), but several adjusted analyses found weak or non-significant associations (26, 33, 35). The evidence therefore supports a possible muscle-strength-to-glycemia signal in prospective studies, whereas the HbA1c-to-strength relationship remains inconsistent.

One complementary cross-sectional study found that HbA1c was independently associated with lower maximal inspiratory pressure, but not with maximum voluntary ventilation (37). Because this outcome reflects respiratory rather than limb muscle strength, it was interpreted separately and was not directly compared with handgrip-strength estimates.

### 3.5 HbA1c and physical performance

Impaired physical performance was reported less consistently but was also clinically relevant. AWGS-based estimates of low gait speed ranged from 5.4% to 40.8% (35, 40, 45). The widest within-cohort difference occurred when AWGS 2014 and AWGS 2019 criteria were compared: low gait speed increased from 13.2% to 40.8% after the threshold changed from <0.8 to <1.0 m/s (45). EWGSOP2-defined low physical performance was 7.4% in one cohort (43), and FNIH-defined low gait speed was 15.0% in another (42). A separate study reported impaired physical performance in 39.3% using SPPB and chair-stand assessments rather than gait speed alone (44). These differences reflect both population characteristics and the outcome test or threshold used.

Only a limited number of lower or moderate-concern studies examined physical performance, and their findings were inconsistent. A longitudinal analysis reported that a reduction in HbA1c of at least 1% was associated with improvement in gait speed but not handgrip strength (28), whereas several cross-sectional analyses did not identify an independent association after adjustment (26, 31, 35). Higher-concern studies reporting positive chair-stand or flexibility results (36) were considered insufficient to establish consistency because the estimates were unadjusted and based on a very small sample.

The evidence for physical performance therefore remains tentative. Performance is a multidimensional outcome influenced by neurological function, cardiovascular reserve, pain, balance, cognition, and behavior as well as skeletal muscle. Current data do not support a consistent HbA1c-physical-performance association.

### 3.6 HbA1c and composite sarcopenia

Composite sarcopenia prevalence varied substantially by diagnostic framework. AWGS-based estimates ranged from 6.3% to 35.8% (26–28, 31, 35, 40, 41, 44–46). FNIH-based reports found prevalence of 12.9% and 16.2% (39, 42). In one cohort, the same data produced an EWGSOP1 prevalence of 16.9% but an EWGSOP2 prevalence of 7.0% (43); another older T2DM cohort reported an EWGSOP2 prevalence of 45.5% (32). Longitudinal data further showed that sarcopenia is dynamic: among initially non-sarcopenic patients, 12.6% developed sarcopenia and 23.8% of initially sarcopenic patients no longer met the criteria after a median follow-up of 36 months (30). A separate cohort reported a prevalence of 6.3% at baseline and 7.8% after one year (28). In several cohorts, individual domain abnormalities were more frequent than composite sarcopenia because a composite diagnosis requires multiple abnormal domains to coexist (40, 44).

Among lower or moderate-concern studies that used adjusted models, findings for composite sarcopenia remained mixed and depended on the diagnostic framework (26, 31, 32, 35, 39, 40, 44, 45). Positive associations were reported in some studies using AWGS or EWGSOP2 criteria (26, 31, 32), whereas other adjusted analyses found no independent association after accounting for age, body composition, nutritional variables, diabetes duration, or complications (35, 39, 40, 44, 45).

When component-specific results were available, positive composite associations were often driven by low muscle mass rather than by impaired strength or physical performance (26, 32). Because AWGS, EWGSOP2, FNIH, and study-specific definitions combine different domains and use different thresholds, prevalence and effect estimates across these frameworks should not be treated as interchangeable or pooled informally. The overall evidence therefore supports diagnostic-criteria-dependent associations rather than a uniform association between HbA1c and composite sarcopenia.

## 4 Potential biological mechanisms

Chronic hyperglycemia and the broader metabolic disturbances associated with T2DM may influence skeletal muscle health through multiple interacting pathways, including impaired insulin signaling and protein homeostasis, advanced glycation end product (AGE) accumulation, mitochondrial dysfunction and oxidative stress, chronic inflammation, neuropathy, and microvascular impairment. These mechanisms represent the biological consequences of sustained hyperglycemic exposure and diabetes-related metabolic abnormalities rather than the effects of HbA1c itself. Therefore, they provide biologically plausible explanations for the observed associations between glycemic control and different skeletal muscle phenotypes. However, given the limited direct human evidence linking specific molecular pathways to specific muscle outcomes, these pathways should be interpreted as overlapping and interconnected mechanistic hypotheses rather than established mediators or causal pathways of the HbA1c–muscle relationship (**Figure 3**).

**Figure 3:**
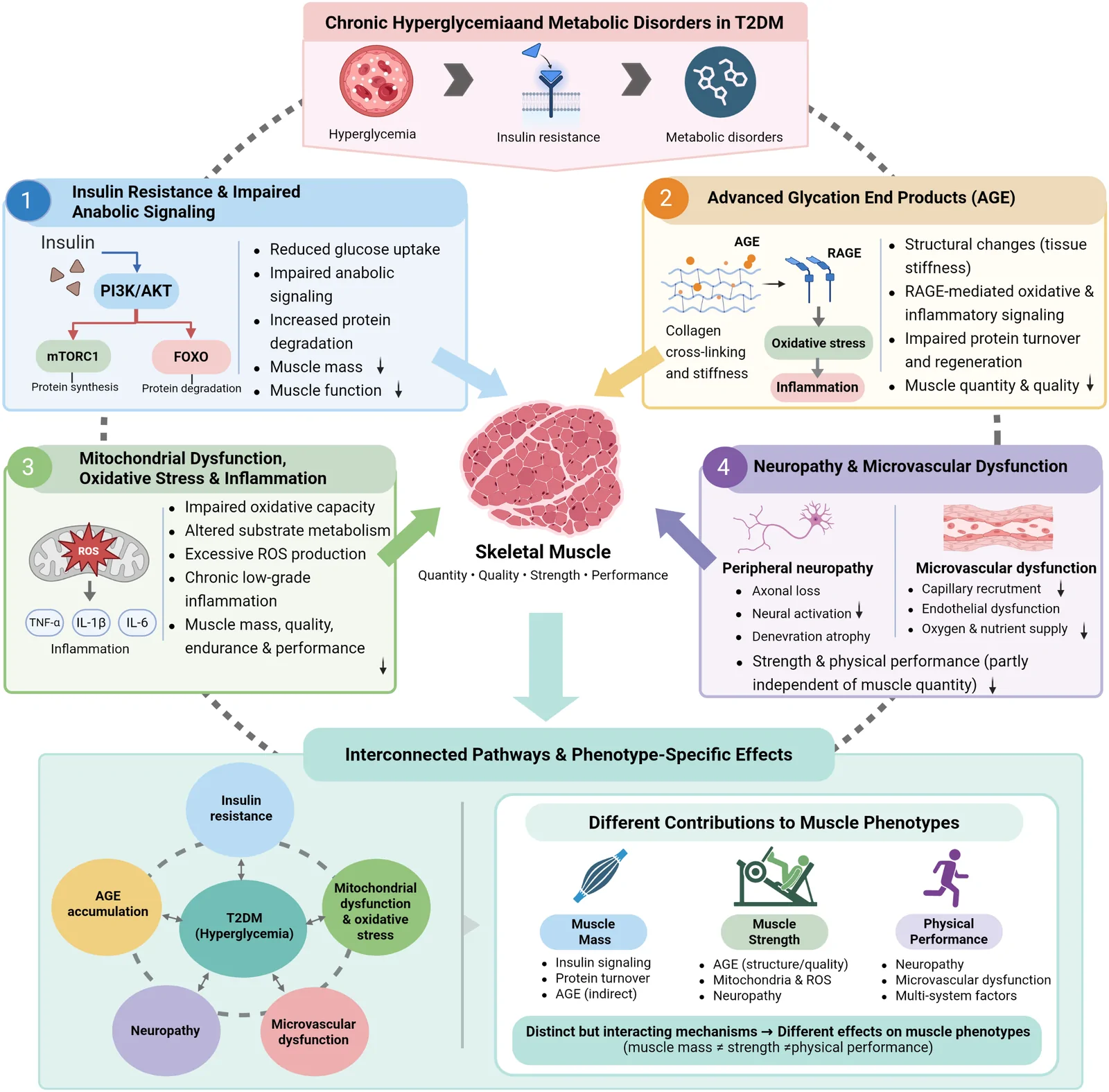
A proposed conceptual framework illustrating potential mechanisms through which chronic glycemic exposure and diabetes-related metabolic dysfunction may influence skeletal muscle phenotypes. This framework depicts hypothesized and overlapping biological pathways rather than established causal relationships. Four interrelated pathway groups are shown: insulin resistance and impaired anabolic signaling, advanced glycation end product (AGE) accumulation, mitochondrial dysfunction with oxidative stress and inflammation, and neuropathy with microvascular dysfunction. Insulin resistance directly impairs protein synthesis and accelerates protein degradation, primarily affecting muscle mass. AGE accumulation induces collagen cross-linking and RAGE-mediated oxidative signaling, contributing to structural changes and impaired muscle quality. Mitochondrial dysfunction reduces ATP production and increases reactive oxygen species, which together with chronic inflammation may influence muscle endurance and fatigability. Neuropathy and microvascular impairment contribute to declines in strength and physical performance through reduced neural activation and diminished oxygen and nutrient delivery. Created with BioRender.com.

### 4.1 Insulin resistance and impaired anabolic signaling

Skeletal muscle is the primary target tissue for insulin to promote glucose uptake and also receives insulin’s anabolic regulation, participating in the maintenance of muscle mass (48). Under physiological conditions, the insulin signal through the PI3K/AKT pathway can promote protein synthesis under the mTORC1-related signaling pathway and inhibit the protein degradation mediated by FOXO (49, 50).

In T2DM, insulin resistance may disrupt this balance by reducing anabolic signaling and promoting protein degradation (51, 52). Since muscle mass directly depends on the balance between protein synthesis and degradation, this disorder may lead to a reduction in muscle mass (53). Clinical studies have suggested that HbA1c is more closely associated with functional muscle outcomes, such as muscle strength and gait speed, than with muscle mass itself, indicating a domain-specific relationship between HbA1c-derived glycemic exposure and sarcopenic phenotypes (27).

However, insulin resistance not only causes a reduction in muscle mass by disrupting protein turnover, but also damages muscle function by affecting glucose uptake and metabolic flexibility (48). Therefore, its impact extends beyond the reduction in muscle mass to include the decline in muscle function.

### 4.2 Advanced glycation end products and muscle quality

Persistent hyperglycemia promotes the formation and accumulation of AGEs in long-turnover structural proteins and connective tissues (54). In skeletal muscle, AGE accumulation may have both structural and signaling effects. Glycation of extracellular matrix proteins such as collagen can increase tissue stiffness and potentially impair force transmission, whereas activation of the AGE receptor RAGE can promote oxidative and inflammatory signaling (55–57).

Higher AGE burden has been associated with lower muscle strength and poorer physical function in observational studies (58, 59), and experimental studies suggest potential adverse effects on protein turnover, cellular homeostasis, and regenerative capacity (60, 61). AGE-related changes may therefore affect muscle quantity as well as muscle quality.

These findings link AGE accumulation to both muscle quantity and quality, but through distinct mechanisms. Glycation-induced collagen cross-linking and increased stiffness within the muscle–tendon unit may impair tissue compliance, force transmission, and ultimately force output, whereas RAGE-mediated inflammatory signaling disrupts protein turnover, mitochondrial function, and cellular homeostasis. Because these biomechanical and molecular pathways may operate independently, AGE accumulation may help explain why changes in muscle quantity and functional performance do not always occur in parallel.

### 4.3 Mitochondrial dysfunction, oxidative stress, and inflammation

Mitochondrial abnormalities are frequently described in insulin-resistant skeletal muscle and may include impaired oxidative capacity, altered substrate metabolism, disturbances in mitochondrial dynamics, and increased generation of reactive oxygen species (62, 63). Reduced mitochondrial efficiency can limit ATP production and contribute to muscle fatigability, while excessive oxidative stress may damage mitochondrial components, contractile proteins, and cell membranes (64, 65).

These abnormalities interact closely with chronic low-grade inflammation. Proinflammatory mediators, including TNF-α, IL-1β, and IL-6, may interfere with anabolic signaling and facilitate muscle protein degradation (66, 67). Oxidative stress and inflammatory signaling can further reinforce one another through pathways such as NF-κB (68).

These processes could influence several muscle phenotypes simultaneously. Catabolic signaling may contribute to loss of muscle mass, whereas impaired energy production and oxidative injury may have proportionally greater effects on muscle quality, endurance, and physical performance. This overlap may help explain some of the heterogeneity in muscle phenotypes observed across clinical studies.

### 4.4 Neuropathy and microvascular dysfunction

Diabetic peripheral neuropathy provides a pathway through which muscle strength and physical performance may deteriorate partly independently of muscle quantity. Axonal loss, impaired nerve conduction, motor-unit remodeling, and reduced neuromuscular activation can decrease force generation and may eventually contribute to denervation-related muscle atrophy (69–71).

Microvascular dysfunction may further impair skeletal muscle function through reduced capillary recruitment, endothelial dysfunction, and diminished delivery of oxygen and nutrients during activity (72, 73). These effects may be particularly relevant to gait speed, endurance, and other measures of whole-body physical performance.

This distinction is important when interpreting the clinical evidence. Physical performance is a multisystem phenotype influenced not only by skeletal muscle but also by neurological function, vascular supply, balance, sensation, cardiovascular reserve, and joint disease. Consequently, HbA1c may show a weaker or less consistent association with gait speed or SPPB than with muscle quantity, even when diabetes-related complications are contributing substantially to functional impairment.

### 4.5 Integrated interpretation across skeletal muscle phenotypes

The mechanisms described above are highly interconnected and should not be assigned exclusively to individual muscle outcomes. Nevertheless, their relative contributions may differ across phenotypes.

Impaired insulin signaling and altered protein turnover provide a relatively direct biological pathway to reduced muscle quantity. AGE accumulation may affect both muscle structure and contractile quality, whereas mitochondrial dysfunction, oxidative stress, and inflammation may influence muscle maintenance, force production, and fatigue resistance. Neuropathy and microvascular impairment may have particularly important effects on strength and physical performance because these outcomes depend on neural activation and systemic physiological reserve in addition to muscle mass. These pathways can also amplify one another. Insulin resistance may promote mitochondrial dysfunction; oxidative stress can enhance inflammatory signaling; AGE/RAGE activation may further increase oxidative and inflammatory stress; and neuropathy or vascular disease may reduce physical activity, thereby accelerating muscle deconditioning.

The mechanistic analysis supports the clinical findings, suggesting that the associations between HbA1c and sarcopenia differ across skeletal muscle phenotypes. This may reflect the fact that muscle mass, strength, and physical performance respond differently to chronic glycemic exposure because they are influenced by distinct but interacting biological mechanisms.

## 5 Discussion

This structured narrative review examined the relationship between HbA1c-derived glycemic exposure measures and skeletal muscle health in adults with T2DM by integrating evidence across distinct skeletal muscle phenotypes and analytical directions. Across the 30 included clinical reports, the available evidence did not support a uniform association between HbA1c and all aspects of skeletal muscle health. Instead, the observed relationships varied according to the phenotype assessed, the direction of analysis, the definition of glycemic exposure, and the methodological characteristics of individual studies.

The risk-of-bias appraisal modified the interpretation of phenotype-specific evidence and influenced how consistency across muscle phenotypes was evaluated. Rather than concluding that muscle mass is intrinsically the most HbA1c-sensitive skeletal muscle phenotype, the current evidence more specifically indicates that the most consistent signal comes from longitudinal analyses examining changes in glycemic exposure and changes in muscle mass among studies rated as having lower or moderate concern. This interpretation is strengthened by the temporal design of these studies but should not be generalized to all study types. In contrast, cross-sectional associations between HbA1c and muscle mass were heterogeneous and were frequently attenuated after adjustment for body composition, diabetes-related factors, and treatment-related variables. Studies relying on unadjusted HbA1c-specific estimates or those with substantial attrition were therefore considered insufficient to independently support evidence consistency. Residual confounding, particularly from antidiabetic therapy, physical activity, nutritional status, body composition, and diabetes duration, remains an important limitation across the current evidence base. Future prospective studies with repeated measurements of glycemic exposure and multidimensional assessment of skeletal muscle phenotypes are needed to clarify the temporal and causal relationships.

### 5.1 Main findings

After considering research quality, exposure definition, and analysis direction, this review concludes four interrelated main findings. First, muscle mass, muscle strength, physical performance, and complex sarcopenia are different dimensions of skeletal muscle health and should not be regarded as interchangeable outcomes; different muscle outcomes may respond differently to physiological stress, which has been demonstrated in models such as bed rest, and in the context of chronic hyperglycemia in T2DM, our findings also suggest that the relationship between blood glucose control and skeletal muscle health varies depending on the evaluated muscle outcome, rather than following a uniform pattern. Second, in the same analysis direction, the most consistent clinical signal is not that muscle mass is inherently more sensitive to HbA1c than other muscle outcomes, but that longitudinal analyses that pay moderate or moderate attention to the changes in HbA1c exposure and muscle mass provide relatively more consistent temporal evidence (6, 28), while the corrected cross-sectional association between a single HbA1c measurement and muscle mass is inconsistent (23, 27, 33, 35). Third, in the opposite analysis direction, the strongest evidence comes from prospective studies, showing that lower baseline muscle strength can predict poorer subsequent glycemic control or a lower likelihood of reaching glycemic targets (7, 17). This direction-specific evidence is consistent with the inter-biological compatibility, but does not establish a bidirectional causal relationship. Finally, the evidence for complex sarcopenia and physical performance further supports the phenotypic specificity and the dependence of the diagnostic framework, with the association with complex sarcopenia varying depending on the AWGS, EWGSOP2, FNIH, or study-specific definitions, and often driven by the low muscle mass component (26, 32), while the evidence for physical performance is still limited, heterogeneous, and insufficient to support a consistent association (26, 28, 31, 35, 36), which is consistent with the multi-system nature of physical performance measurement.

### 5.2 Interpreting phenotype heterogeneity and directionality

The phenotype-dependent findings are consistent with the biological complexity of skeletal muscle, but methodological factors also contributed substantially. Muscle mass, strength, and performance capture different aspects of muscle structure and function and were measured with different instruments and thresholds (13, 74). More importantly, the strength of the observed associations differed according to whether HbA1c was analyzed as a single continuous value, a categorical threshold, a longitudinal change, or a treatment target. Therefore, the evidence does not justify the conclusion that one phenotype is intrinsically more susceptible to HbA1c than another.

Evidence from type 1 diabetes mellitus (T1DM) provides additional context for interpreting these findings. Although the present review focused on T2DM, sarcopenia and impairment of individual muscle domains have also been reported in T1DM, suggesting that chronic dysglycemia may represent a shared contributor to skeletal muscle deterioration across diabetes phenotypes (75, 76). However, the metabolic background differs substantially between T1DM and T2DM, with T2DM being more strongly characterized by insulin resistance, obesity, and altered body composition. Therefore, muscle abnormalities observed in diabetes may reflect both common consequences of prolonged hyperglycemic exposure and disease-specific metabolic factors. These findings further support the interpretation that HbA1c captures only one component of the complex determinants underlying skeletal muscle health rather than a comprehensive marker of muscle impairment.

Beyond differences in phenotype and diabetes type, the direction of analysis also requires careful interpretation. Studies examining HbA1c as a predictor of later muscle outcomes address one temporal question, whereas studies examining muscle characteristics as predictors of later glycemic control address the opposite question. Both directions produced some evidence, but the studies did not evaluate the same biological mechanism. The results are therefore described as direction-specific and potentially reciprocal, rather than as proof of a bidirectional biological relationship.

Residual confounding may also explain part of the apparent phenotype heterogeneity. Nutritional status remains an important source of residual confounding. Although ten reports directly assessed at least one nutritional variable, only four quantified energy or protein intake, and nutritional variables were rarely retained in the models used to estimate HbA1c-muscle associations. The most frequently available nutrition-related variable was serum albumin; however, albumin reflects both nutritional status and systemic inflammation and is not a substitute for quantitative dietary assessment. Adjustment for antidiabetic treatment, weight and body composition, physical activity, nutritional status, and diabetes duration varied across studies. Higher-concern studies, including those with unadjusted HbA1c-specific estimates or substantial attrition, were not used alone to support evidence consistency.

### 5.3 Comparison with previous evidence

Previous systematic reviews and meta-analyses have generally examined sarcopenia as a composite outcome and reported an overall association with poor glycemic control (10, 14, 74). The present review extends that literature by separating individual muscle domains, distinguishing HbA1c exposure constructs, and incorporating design-specific risk-of-bias assessment. After these steps, the evidence is less consistent than a simple pooled association would suggest.

The strongest quality-informed signal was observed for longitudinal HbA1c change and change in muscle mass among lower or moderate-concern studies (6, 28). Cross-sectional findings were more heterogeneous, and composite sarcopenia results varied by diagnostic framework (26, 31, 32, 35, 39, 40, 44, 45). Thus, the present synthesis supports a more specific conclusion than previous reviews: the consistency of evidence depends on the exposure construct, muscle phenotype, analytical direction, and study quality.

### 5.4 Clinical implications

The clinical implication is not that HbA1c should be used as a diagnostic or surrogate marker for sarcopenia. Rather, satisfactory HbA1c should not be interpreted as excluding sarcopenia or impairment in an individual muscle domain. Conversely, poor glycemic control may identify patients who warrant closer attention to muscle health, but HbA1c alone cannot determine the presence, type, or severity of muscle impairment. Muscle mass, strength, and physical performance should therefore be assessed directly using validated measures when clinically indicated.

Management should address muscle health alongside glycemic control. Physical activity, resistance training, adequate energy and protein intake, and treatment of diabetes-related complications may influence muscle outcomes independently of HbA1c. Because treatment-related weight loss, nutrition, and physical activity were inconsistently measured and adjusted for, the available observational evidence does not establish that modifying HbA1c alone will improve muscle mass, strength, or physical performance. Although some studies incorporated nutritional indicators into multivariable models, quantitative assessment of energy intake, protein intake, and dietary patterns was rarely available, limiting the ability to determine their independent contribution to skeletal muscle outcomes. This consideration is particularly relevant for glucose-lowering therapies that influence body weight or energy balance, as treatment-related changes in lean mass may contribute to observed associations between HbA1c trajectories and muscle outcomes.

### 5.5 Strengths and limitations

This study employs phenotypic stratification and direction-specific methods to investigate whether there are differences in the relationship between HbA1c-derived glycemic exposure measures and muscle mass, strength, and physical function. By categorizing studies according to the direction of analysis, we distinguish between evidence from studies that use HbA1c as a predictor of muscle outcomes and evidence from studies that use muscle characteristics as predictors of glycemic control. Although these two types of questions are related, they are distinct; confusing them may obscure meaningful differences. The evidence table preserves key study details, including the definition of HbA1c, methods of phenotypic assessment, strategies for adjusting for covariates, and limitations noted by the original authors, enabling readers to independently evaluate the results of each study without relying solely on our summary.

This review also has certain limitations. The included studies were primarily observational, with a significant proportion being cross-sectional studies, which precludes causal inferences. Although longitudinal studies are suitable for exploring temporal associations, they are susceptible to residual and time-varying confounders such as body weight, physical activity, nutritional status, disease severity, and treatment changes over time. In addition, there was considerable methodological heterogeneity among the studies. HbA1c measurements employed different thresholds, were assessed as continuous variables, or analyzed based on longitudinal changes; muscle outcome measures were evaluated using BIA, DXA, or CT; and the definitions of sarcopenia in older adults varied among the EWGSOP, AWGS, FNIH, and study-specific criteria, limiting the feasibility of direct comparisons. An additional limitation is that this review focused specifically on HbA1c-derived glycemic exposure measures and did not include continuous glucose monitoring (CGM)-derived metrics. Although HbA1c reflects average glycemic exposure over the preceding approximately 2–3 months, it does not capture short-term glycemic fluctuations, time in range, time above or below range, or hypoglycemic exposure. Therefore, CGM-derived parameters may provide complementary information regarding the relationship between dynamic glycemic patterns and skeletal muscle phenotypes, and future studies integrating HbA1c with CGM metrics are warranted. Treatment-related confounders are another concern, as antidiabetic medications may affect HbA1c but may also influence body weight, appetite, body composition, and muscle metabolism through mechanisms unrelated to glycemic control. Therefore, the association between changes in HbA1c and muscle outcomes should not be interpreted as a direct effect of antidiabetic therapy. Some reports originate from overlapping cohorts, particularly the MUSCLES-DM study; multiple published results from the same cohort do not constitute independent replication, which may lead to an exaggerated impression of the evidence base. Finally, although design-specific JBI critical appraisal was completed and used to weight the interpretation of evidence, no quantitative meta-analysis was performed. The conclusions therefore represent a structured, quality-informed narrative synthesis rather than precise pooled estimates.

### 5.6 Future research directions

Future research can be advanced in the following aspects. First, a prospective design should be adopted to repeatedly measure blood glucose exposure and multi-dimensional skeletal muscle outcomes for the same subject, in order to determine whether the changes in each indicator are synchronous. At the same time, the exposure and outcome definitions should be standardized, and specific outcomes in each muscle domain as well as detailed information on drugs, physical activity, nutrition, body composition, and complications should be reported. Second, clinical phenotypes and mechanism studies should be combined. Future research can simultaneously collect biological samples and skeletal muscle assessment data in longitudinal cohorts, including comparisons across different diabetes phenotypes, to determine whether pathways such as insulin resistance, AGE accumulation, inflammation, and neuropathy contribute differently to specific muscle outcomes. Third, intervention trials should evaluate whether improving blood glucose, increasing physical activity, or adjusting nutrition can improve muscle mass, strength, or function, and distinguish the effects of metabolic improvement and specific treatments (especially hypoglycemic drugs that affect weight and muscle). Fourth, attention should be paid to subgroups such as the elderly, obese, long-term patients, neuropathy, or microvascular complications. Identifying these modifying factors is more clinically significant than seeking a single HbA1c threshold applicable to all patients.

## 6 Conclusions

This structured narrative review suggests that the relationship between glycemic control and skeletal muscle health in T2DM is phenotype-specific rather than uniform across muscle mass, muscle strength, physical performance, and composite sarcopenia. The observed associations vary according to study design, analytical direction, phenotype definition, and clinical context, and current evidence does not establish bidirectional causality. HbA1c should be interpreted as a marker of glycemic exposure rather than an indicator of overall skeletal muscle health. Importantly, satisfactory glycemic control does not exclude the presence of sarcopenia or impairments in individual muscle domains. Therefore, muscle health should be directly assessed using validated approaches when clinically indicated, independent of glycemic status. Future prospective and interventional studies using repeated glycemic measurements, detailed assessment of antidiabetic treatment exposure, lifestyle factors, nutritional status, body composition, and standardized domain-specific muscle assessments are needed to clarify temporal relationships and clinical relevance.
